# The Responses of Young Domestic Horses to Human-Given Cues

**DOI:** 10.1371/journal.pone.0067000

**Published:** 2013-06-19

**Authors:** Leanne Proops, Jenny Rayner, Anna M. Taylor, Karen McComb

**Affiliations:** Mammal Vocal Communication and Cognition Research, Department of Psychology, University of Sussex, Sussex, United Kingdom; University of Rennes 1, France

## Abstract

It has been suggested that the process of domestication, at least in some species, has led to an innate predisposition to be skilled at reading human communicative and attentional cues. Adult domestic horses (*Equus caballus*) are highly sensitive to subtle bodily cues when determining if a person is attending to them but they are less adept at using human communicative cues in object choice tasks. Here we provide the first study into the ontogeny of such skills in order to gain insights into the mechanisms underlying these abilities. Compared with adult horses, youngsters under the age of three could use body orientation but not more subtle cues such as head movement and open/closed eyes to correctly choose an attentive person to approach for food. Across two object choice experiments, the performance of young horses was comparable to that of adult horses – subjects were able to correctly choose a rewarded bucket using marker placement, pointing and touching cues but could not use body orientation, gaze, elbow pointing or tapping cues. Taken together these results do not support the theory that horses possess an innate predisposition to be particularly skilled at using human cues. Horses' ability to determine whether humans are attending to them using subtle body cues appears to require significant experience to fully develop and their perhaps less remarkable use of limited cues in object choice tasks, although present at a much earlier age, is likely to reflect a more general learning ability related to stimulus enhancement rather than a specific ‘human-reading’ skill.

## Introduction

The ability to use the attentional and communicative cues of others allows animals to gain important information about the world around them. For many domesticated and captive animals humans represent significant social partners and sources of information. It is clear that many captive wild animals are able to learn to read human behaviour through exposure to people during their lifetimes [1], [2]. However it has also been suggested that, through the process of domestication, some species may have been specifically selected for an ability to use human communicative and attentional cues in functionally relevant ways [3]. If a species has a predisposition to be skilled at using human cues, the skill is likely to be present from a very early age, requiring minimal exposure to humans to develop fully. Support for the domestication hypothesis in dogs (*Canis familiaris*) comes from the finding that very young puppies are skilled at reading human communicative cues [4], but see [5]. However, this ability has yet to be systematically studied in the young of any other domesticated species. Thus the aim of this study is to assess the extent to which young horses are able to use human communicative cues in two standard tests and compare their performance to that previously reported for adult horses. The results will therefore provide insights into the role of heredity and experience in the development of these abilities in a second domestic animal species.

Our first experiment investigates the ability of young horses to use human attentional cues. A large number of species (e.g. goats (*Capra hircus*) [6]; rhesus macaques (Macaca mulatta), [7]; gibbons (*Hylobates agilis*) [8]; great apes, [9]; dolphins (*Tursiops trancata*) [1]; dogs [10]; ravens (*Corvus corax*) [11]; tortoises (*Geochelone carbonaria*) [12]; horses (unpub. data) are able to follow the gaze of others and more complex gaze following studies suggest that some species such as apes, dogs and certain corvids have a good understanding of the relationship between seeing and knowing [10], [11], [13]–[15]. Other paradigms show that some animals are sensitive to subtle human eye cues in a competitive context, e.g. [16], [17], [18] and dogs and horses are also able to use eye cues when deciding whether to obey a command [19], [20]. The use of attentional cues such as the presence of eyes, or schematic representations of eyespot patterns, evoke anti-predator behaviour in many species and can be present from a very early age [21], [22]. The intensity of an animal's reaction to approaching humans can depend, not just on body orientation but the direction of head and the visibility of eyes [23]–[25]. It is presumed that this type of behaviour is triggered by a simple reflexive eye detector mechanism yet the ability to detect eye direction and attribute attention in a social context has been considered to be a phylogenetic and ontogenetic precursor to possessing a theory of mind [7], [26]–[28]. These very different interpretations of attention attribution highlight the importance of determining the underlying cognitive mechanisms involved in utilising another individual's postural and communicative cues and the contexts in which they are used.

Although the ability to detect and follow gaze in competitive or predator-prey contexts is widely reported, many species seem to have more difficulty using attentional cues in a cooperative, social context and rely on head orientation rather than more subtle eye cues in this situation [29]–[31]. When preferential looking and competitive food paradigms are employed, many primate species show sensitivity to subtle eye cues that, in some cases, develops at an early age [8], [17], [32]–[34]. Chimpanzees also readily adjust their begging behaviour in response to the attentional cues of experimenters, giving more visual cues when they are attending to them and more auditory cues when the person is inattentive [35]. However, results from ape studies using the same cooperative, attention attribution paradigm as the one employed in this study have been mixed, with some experiments showing apes can readily use subtle eye cues [36] and others not [27], [37]–[39]. In this task subjects have to decide whom to approach to receive food when presented with two people, one attentive and one inattentive. In this context both adult horses and domestic dogs reliably use subtle human attentional cues such as open versus closed eyes, without any explicit training [10], [19], [40]–[43].

To date no one has administered the specific attention attribution task presented in this paper to young subjects of any domestic species. However, one study of 2 year-old horses with minimal prior exposure to humans did assess their sensitivity to attentional cues in a learned, obedience task. Following the training phase, subjects obeyed a command to ‘stay’ regardless of the attentional state of a familiar handler but obeyed more readily when a stranger giving the command was paying attention to them [20]. They were sensitive not only to the body orientation of the stranger but also their eye direction, suggesting that young horses are also capable, in some contexts, of attending to very subtle human cues to attention. The aim of our experiment is to assess whether young horses are able to spontaneously use human attentional cues without any direct training and to compare their performance to that of adult horses tested in our previous study [43]. We therefore systematically varied the type of attentional cues presented to determine, if subjects could use human attentional cues, whether they used gross, approximate cues such as body orientation and head orientation or the most accurate cue to attention, the eyes. We also included a mixed cue trial where the head and eye cues were conflicting. If the subjects were capable of using head and eye cues, this condition would assess whether the crucial cue of eye direction was more salient than that of head orientation.

The second task given to young horses in this study was a standard task used to assess the ability of animals to read human cues, the object choice task. Here subjects are presented with a choice of two or three containers and a person directs their attention towards the rewarded container using a variety of communicative cues. Cues that have been tested in this task include gazing, tapping, markers and a wide variety of pointing cues including those that are close to the target (proximal points), those that are further away (distal points), those that are present when the choice is made (sustained points) and those that are only given for a short time before the choice is made (momentary points); points have also been given across the body and with different parts of the body including the leg and elbow [44]. Chimpanzees begin to use human-given cues in the object choice task around 11 months of age [45]. Apes and other primates often perform surprisingly poorly in this task, although whether this is due to a lack of motivation, a methodological artefact or reflects a genuine lack of ability remains unclear [46]–[51]. What is clear is that dogs perform very well in this task and are able to use a wide variety of human given cues. Although cues that protrude from the human's body and are closer to the container are more salient, with, for example, elbow pointing being less informative than pointing with the whole arm [52], dogs are also able to use gazing and momentary pointing at a distance from the target, both of which do not involve any form of local enhancement [53]. Debate continues regarding the extent to which this ability is learnt through exposure to humans. There is some indication that adult feral and shelter dogs perform less well than pet dogs, suggesting enculturation may be important, yet very young puppies are able to use a variety of cues and tend to outperform hand reared wolf pups, suggesting that dogs have a predisposition to be skilled in this task [4], [5], [54]–[56]. Other domestic animals that have been tested in the object choice task include domestic goats, with minimal exposure to humans, that were able to use a distal sustained point and touch cue [6]. Domestic cats perform at a level comparable to domestic dogs in object choice tasks and are able to use the most challenging point cue – the distal momentary point [57]. The results of a comparison between 2 wild boar groups (*Sus scrofa scrofa*) and 2 domesticated pig groups (*S. s. domestica*) living in either enriched or more impoverished environments suggest that experience during a subject's lifetime rather than their domestication status is the best predictor of success in this task [58]. However, to date only the young of domestic dogs and wolves have been tested.

In object choice tasks, adult horses do not perform as well as dogs but have been successful in using a number of cues including the presence of a human, a marker cue and pointing cues if the cue is close to the container or if the cue is sustained during the choice. However, they are not able to use the more difficult distal momentary pointing cue nor gaze or body orientation cues [59]–[61]. This pattern of results coupled with the observation that subjects often approach the outstretched hand or marker before investigating the bucket, has led to the conclusion that horses, like goats, are able to use cues that provide stimulus enhancement but they do not have an understanding of the communicative intent behind the cues. It must be noted however, that our previous research found that horses are able to use a proximal momentary pointing cue but do not use a highly salient momentary tapping cue, for possible explanations of this result see [60], findings which are not totally compatible with the hypothesis that horses only use stimulus enhancement cues. In the experiments reported here we included a wide variety of cues providing varying degrees of stimulus enhancement, either during or prior to when the choice was made, in order to further elucidate the mechanisms used by horses in this task. Strong stimulus enhancement is provided by the touching and marker placement cues, although in the case of the marker cue, an object rather than the experimenter provides the enhancement at the time the choice is made. The sustained pointing cue provides reasonably strong enhancement, the elbow point cue less so, and the body orientation and gaze alternation cues provide no direct stimulus enhancement cues, requiring the subject to infer where the attention of the experimenter is being directed. Thus the aim of our two object choice experiments in the current study is to compare the performance of young horses to that we reported previously for adult subjects. In addition we provide data on young horses' ability to use a variety of touching/tapping cues in order to try to clarify the mechanisms by which horses use certain cues and to try to determine why the adult horses tested previously did not use a seemingly basic momentary tapping cue.

Taken together, the three experiments here provide further insights into the domestication hypothesis by testing the young of a another domesticated species, other than dogs, on tasks involving the use of human cues. If young horses are not as adept as adult horses at using human attentional and communicative cues, this will suggest that extensive learning during their lifetime is required and that these abilities may not have been strongly selected for during the domestication of horses.

## Methods

### Ethics Statement

The method employed in this study involved interactions that were similar to those the horses were likely to experience in their normal daily routine. Trials were carried out in a familiar setting. The data recorded was observational and non-invasive and as such this study did not require a licence under the United Kingdom Home Office regulations concerning animal research and welfare. This study complied with the University of Sussex regulations on the use of animals and was approved by the School of Psychology ethics committee. No subjects showed signs of stress during the trials.

### Subjects

A total of 35 young horses under the age of three participated in this research, 22 subjects completed the attention attribution task, 25 subjects completed the first object choice task and 15 subjects completed the second object choice task. The attention attribution task included 12 males and 10 females, and ages ranged from 6 months to 2.8 years (*X* ± S.E.  = 1.80±0.19). The first object choice task included 13 males and 12 females, and ages ranged from 9 months to 2.9 years (*X* ± S.E.  = 1.75±0.16) and the second object choice task included 8 males and 7 females with ages ranging from 4 months to 3 years (*X* ± S.E.  = 2.06±0.21), see Table 1 for subject details. For those that completed the attention attribution task and the first object choice task, order was counterbalanced across subjects. Some subjects also participated in the second object choice task, which was conducted more than 6 months after the other two tasks were completed. Subjects were from 9 locations and were either privately owned or were from stud farms. Since these tasks required the youngsters to be halter led for trials that lasted on average 10–20 minutes, most of the subjects had been regularly handled. Subjects were not food deprived prior to the study.

**Table 1:** Subject details and scores on the tasks completed.

Name	Age at test	Sex	Location	AA task.		OCT task 1.		OCT task 2.	
				Scores & cues used		Scores & cues used		Scores & cues used	
BKM	2.75, 2.5	M	WDN	2	B H	5	P E T B G		
RCA	2, 2	M	IKN	4	B H E M	4	E T B G		
ERN	2.5, 2.5	M	MNL	2	B E	3	P E G		
NLA	0.75, 0.75	F	TWY	1	B	3	P E T		
GTR	0.75, 0.75	M	TWY	1	M	2	P B		
NTW	0.75, 0.75	M	TWY	3	B E M	2	P E		
SAF	2.75, 2.75	F	WRF	3	B E M	3	E T B		
RUB	2.75, 2.75	M	WRF	3	B H M	3	P T G		
LIA	2.5, 2.5	M	RSC	2	B H	1	P		
JES	0.75, 2.5	F	WDN	4	B H E M	3	P E G		
SPR	0.8, 1, 2.25	F	WDN	3	B H E	3	P T G	3	M G A
HPT	1.7, 2, 3	F	WDN	0		2	E T	3	M G T
GZR	2.8, 2.9	M	WDN	3	B H E	3	P E T		
PPT	2	F	WDN	3	B E M				
WLW	2.8, 1.5	F	WDN	1	B	3	T B G		
PNC	1.9, 2, 2.9	M	WDN	3	B H E	4	P E B G	3	M T A
FLK	1, 0.9, 1.9	M	WDN	2	B H	2	P E	4	M G T A
POP	2.75, 1.5	F	WDN	1	H	2	E G		
SAM	1, 0.9, 1.8	M	WDN	2	E M	3	E T G	3	G T A
APL	2.8, 2.8	F	WDN	2	B M	4	P E T B		
MDN	1.25, 1.2	F	WDN	2	B H	2	P E		
TDY	0.5, 1.25	M	WDN	2	E M			3	G T A
MAY	1.5	F	MLH			2	B G		
DVC	0.75	F	TWY			3	P B G		
DEF	0.75	M	TWY			4	P T B G		
RGG	2.9	M	WDN			2	P B		
RGS	1.5, 2.3	F	WDN			2	P E	3	G T A
IMG	0.4	F	WDN					3	M G T
GHS	1.2	F	WDN					3	M T A
RIO	2.9	M	WDN					4	M G T A
BRZ	3	M	WDN 2					4	M G T A
MSY	2.8	F	WDN					2	M G
IDY	2	F	WDN					4	M G T A
BDY	1.2	M	CHY					3	M T A
OTO	1	M	CHY					3	M G A

Where subjects were tested on multiple tasks, ages at the time of each task are given in the order the tasks appear in this table. Abbreviations for cues correctly used by the subjects: *Attention Attribution (AA) task:* B – Body orientation; H – Head orientation; E – Eyes open/closed; M – Mixed cue. *Object choice (OCT) task 1:* P – Point; E – Elbow point; T – Tap; B – Body orientation; G – Gaze/Head alternation. *Object choice (OCT) task 2:* M – Marker; G – Sustained touch gazing at ground; T – Momentary touch gazing at ground; A – Sustained touch gazing ahead.

### Procedure

Subjects were tested in an area familiar to them, either an indoor or outdoor school or an outdoor paddock. One young foal that had not yet been weaned was tested in its own field with its mother and other youngsters and mares in the field held nearby. Trials were conducted between November 2008 and June 2012. Prior to testing, subjects were given a food preference test to see what reward should be given during the trials – choices were between carrots, commercial horse treats and the subjects' normal feed. A number of the young horses had small teeth and had never eaten carrots or treats before so were given their normal feed. All experimenters and handlers were female.

### Attention attribution task

In this study we replicated the general procedure of Proops & McComb [43] using foals and juvenile horses rather than adults. Subjects were presented with two people, one that was paying attention to them and one that was inattentive. Horses were released to determine whom they chose to approach to receive food.

**Figure 1:**
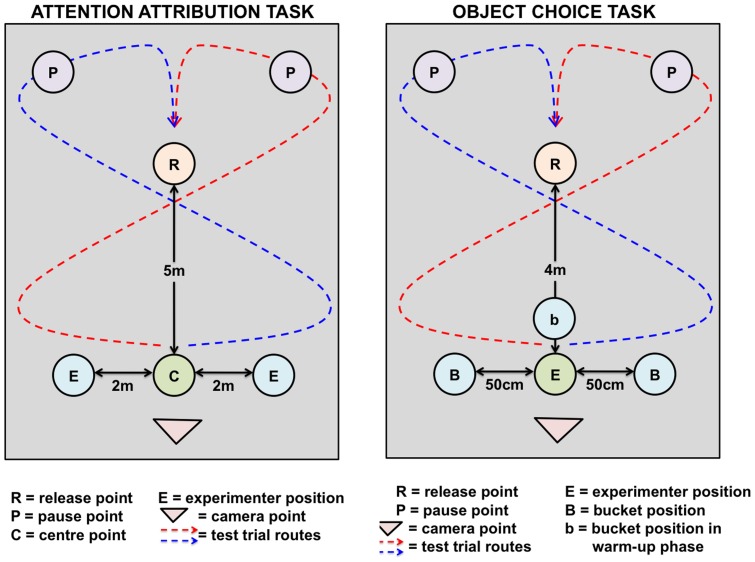
Diagrams of the experimental set-up for a) the attention attribution task and b) the object choice task.

The experimental set up can be seen in Figure 1A. 10 subjects were given a warm up phase in which the experimenters were attentive and 12 were given a warm up phase where the experimenters were inattentive. This was to ensure that the horses were not choosing experimenters in the test phase based on any attentional cues learnt during the warm up phase. It also replicates the protocol of the previous attention attribution study conducted with adult horses. In the attentive warm up the two experimenters stood at centre point C facing the subject with their hands outstretched together holding a food reward. In the inattentive warm up phase the two experimenters stood at 90° to the subject, facing each other at centre point C with their hands outstretched in the middle of them holding the reward. The handler held the subjects on the left side on a loose lead rope and led them towards the centre point to receive their reward. The subjects were then led in a semi-circle to the left or the right (the order was counter balanced to prevent side bias) and the procedure was repeated. The experimenters also swapped sides between each warm up trial to prevent any side biases occurring. The subjects' behaviour was gradually shaped over a maximum of 10 trials so that by the end of the warm-up phase the handler was able to lead to horse to the release point (R), remove the lead rope and the subject would move forward to the experimenters to receive the reward. If after 10 warm-up trials the subjects were still not walking forward to receive a treat when released at the release point then they were excluded from the test trials.

The test phase was the same for all subjects. Four cues were presented to the subjects in a counterbalanced order with an additional reinforcement trial between each test trial. After the warm up phase was complete, the two experimenters moved to points E (Figure 1A) and adopted either an attentive or inattentive stance. The side of the attentive person, the identity of the attentive person and the side the experimenters stood on was counterbalanced across trials. Horses were not given a reward during a test trial but all subjects readily approached an experimenter. The subjects were led in a large circle and walked several meters down the middle line with their heads always oriented forward before they were released at the release point to ensure they had both of the experimenters in their field of vision for a significant amount of time prior to making their choice. Once the horse had approached an experimenter in the test trial, the handler collected the subject and the experimenters returned to centre point C and jointly offered a food reward while they both either adopted the attentive or inattentive pose used in the warm-up trials. Subjects were led in a semi-circle and rereleased at point R to receive a reward. This reinforcement trial was therefore the same as a warm-up trial and was found to increase the motivation of the subjects and improve response rates in adult horses. Subjects were then led in a figure of eight across the test area and held for approximately 30 seconds at point P. This was found to reduce side bias in adult horses. If a horse failed to respond to a cue, a reinforcement trial was given and the cue repeated a total of three times. If the subject still failed to respond to the cue, a recording of “no response” was made and the next cue was presented. Of the 22 subjects, 2 subjects failed to choose an experimenter for three consecutive body cue trials and one subject failed to respond to the eye cue and so they were given a “no response” score for that cue type.

#### Four cues to attention were tested

For the body orientation condition the inattentive person stood with their body turned 180° away from the subject. In the head orientation condition they stood with their body facing forwards but their head turned 90° away (also facing away from the other experimenter), and in the eyes closed condition they stood facing forwards but with their eyes closed. During these three trials the attentive person stood facing forwards and maintained eye contact with the subject while keeping their head still. A fourth, mixed condition was included where the attentive person stood with their head facing towards the ground but their eyes looking up towards the subject while the inattentive person stood with their head facing forwards towards the subject but their eyes looking down towards the ground. In this condition we contrasted head and eye cues to assess whether young horses prioritised eye cues when they conflicted with head cues. The mixed cue given to adult horses in the previous experiment presented conflicting body and head cues. Neutral facial expressions were adopted throughout the trials.

### Object choice task

In the following two experiments we replicated the general procedure of Proops et al [60] using foals and juvenile horses rather than adults. An experimenter cued one of two buckets and subjects were released to determine which bucket they chose to approach.

The experimental set up can be seen in Figure 1B. During the warm up phase the experimenter stood at point E with two black buckets (40 cm diameter, 19 cm height) stacked together in front of them at point b. Food was placed in the bucket and the handler led the subject from the left side on a loose lead rope towards the bucket to collect the reward. The subject was then led in a semi-circle to the left or right and returned to the centre line and was again led towards the bucket to receive the reward. The experimenter swapped the buckets over between each warm-up trial so that each bucket would smell of the reward. The behaviour of the subject was gradually shaped over a maximum of 10 trials so that by the end of the warm up phase the subject could be released at point R and would walk to centre point b and receive the reward from the bucket. If, after 10 warm-up trials, the subject was not walking forward to receive a treat when released at the release point, they were excluded from the test trials.

After the initial warm up phase the experimenter placed the two buckets at points 50cm to the left and right of point E. As the horse approached the release point along the centre line the experimenter gave a cue towards one of the buckets. Again the subjects were led in a large circle and walked several meters down the middle line with their heads always oriented forward before they were released to ensure they had the experimenter in their field of vision for a significant amount of time and would have witnessed the presentation of the cues in every trial. The subject was then released and if the cued bucket was chosen, a food reward was placed in the bucket as soon as the choice was made. Food was not placed in the bucket prior to the choice being made to prevent sight or odour cues affecting the choice. After the test trial the experimenter returned the buckets to the centre point and the horse was led in a semi-circle and re-released at point R to receive a reward. This reinforcement trial was found to reduce side biases and improve response rates in adult horses. Subjects were then led in a figure of eight across the test area and held for approximately 30 seconds at point P. This was found to reduce side bias in adult horses. If a horse failed to respond to a cue, a reinforcement trial was given and the cue repeated a total of three times. All subjects responded to the cues in these tasks within three attempts.

In the experiment in which five cues were presented, the side of the cue was counterbalanced across subjects with half receiving three cues to the left and half receiving three to the right. In the experiment in which four cues were presented, each subject received 2 cues to the left and two to the right. The side to which the cues were given was pseudo-randomised with the constraint that the same side was not cued more than twice in a row. The order of cue presentation was counterbalanced across trials with each cue being presented first, second, third, fourth (and fifth) an equal number of times.

In the first object choice experiment five cues were given:

1. Distal sustained pointing cue: When the subject was approximately 1 m from the release point the experimenter brought her ipsilateral arm out from the side of her body to point towards one of the buckets. This position was held with the body oriented forwards, looking directly ahead until a choice was made. The index finger was approximately 65 cm from the top of the bucket.
2. Sustained elbow point cue: When the subject was approximately 1m from the release point the experimenter brought her elbow out to one side with her hand held on her chest. This position was held with the body oriented forwards, looking directly ahead until a choice was made. The elbow was approximately 105 cm from the top of the bucket.
3. Momentary tapping cue: When the subject was approximately 1 m from the release point the experimenter reached towards the correct bucket and tapped the side slowly three times with large movements of the arm. She then returned to a standing posture, body oriented forwards, looking directly ahead until a choice was made.
4. Sustained body orientation cue: As the horse approached the release point the experimenter turned her whole body towards the correct bucket and stood looking down at the bucket until a choice was made.
5. Sustained gaze alternation cue: Keeping her body oriented forwards, the experimenter alternated the direction of her head and gaze between the horse and the correct bucket until a choice was made.

In the second object choice test 4 cues were given:

1. Marker placement: When the subject was approximately 1 m from the release point the experimenter placed a blue and yellow striped wooden block (18.5×7×3.5 cm) in front of, and touching, the correct bucket. The experimenter then returned to an upright position facing forwards, looking directly ahead.
2. Sustained touching, gazing ahead: When the subject was approximately 1 m from the release point the experimenter bent down and held the side of the correct bucket while gazing ahead towards the subject.
3. Sustained touching, gazing at the ground: When the subject was approximately 1 m from the release point the experimenter bent down and held the side of the correct bucket while gazing at a point on the ground 1 m in front of her.
4. Momentary touching, gazing ahead: When the subject was approximately 1 m from the 4. release point the experimenter bent down and touched the side of the correct bucket and then returned to an upright position facing forwards looking directly ahead.

### Behavioural and statistical analysis

Responses were recorded using a Sony digital handycam video recorder and coded by two independent experimenters; there was no discrepancy between the experimenters in their coding of correct and incorrect choices. For the attention attribution task, the dependent variable was whether the subjects correctly chose the attentive person over the inattentive person when determining whom to approach to receive food. A choice was defined as correct if the subject stood within 1 meter of the attentive experimenter within 60 seconds of being released. In the attention attribution task results from the groups given different warm up phases were compared using 2×2 Fisher's Exact tests. In the object choice task the dependent variable was whether they chose the cued bucket. A choice was recorded as correct if the subject's head approached within 20 cm of a bucket within 60 seconds of being released. In most trials subjects touched the chosen bucket but in some cases subjects looked into the bucket without touching it.

In each task, the number of subjects choosing the correct target for each trial type was analysed using two-tailed binomial tests (where N is the number of subjects and K represents the number of correct responses). For each cue that had previously been given to adult subjects under the same protocol in the studies Proops & McComb [43] and Proops et al. [60], the performance of the young horses was compared to that of adult horses using 2×2 Chi Square tests or Fisher's Exact tests when >80% of expected cell frequencies were less than 5. The total number of correct scores was calculated for each subject in each task and effects of sex analysed using a Mann-Whitney U test. The proportion of right side choices made by each subject was calculated and overall side preferences were assessed using one-sample t tests. “No responses” were excluded from all analyses and total scores were calculated as a percentage of the total number of trials in which a choice was made.

In the attention attribution task and first object choice tasks, in which the samples sizes were large enough to allow further analysis, subjects were also divided into 3 age groups (up to 1 year inclusive, up to 2 years inclusive, up to 3 years) and the effect of age on total scores was analysed using a Kruskall-Wallis test. Performance of subjects on individual cues according to age was also assessed using 2×3 Fisher's Exact tests. We also wanted to ensure that the smaller subjects were equally likely to use the cues provided, particularly in trials where facial cues were important. To assess whether there was an effect of size, subjects were divided into three size categories: subjects with a wither height under 115 cm, subjects 115–130 cm and subjects over 130 cm. Performance of subjects on individual cues according to size was assessed using a 2×3 Fisher's Exact tests and the effects of size on overall scores was assessed using a Kruskal-Wallis test. Fisher's Exact tests were run at the VassarStats website: http://faculty.vassar.edu/lowry/VassarStats.html. All other statistical analyses were performed using SPSS v. 17.0.0 software for Mac.

## Results

The results for each individual subject across all the tasks they completed can be seen in Table 1.

### Attention attribution task

There were no significant differences in the performance of the subjects given the attentive or inattentive warm up phase for any cue type, suggesting that their responses were not conditioned by specific cues given during the warm up phase (Fisher's Exact Tests: body cue: *N* = 20, *P* = 0.22; head cue: *N* = 22, *P*>0.99; eye cue *N* = 21, *P* = >0.99, mixed cue: *N* = 22, *P* = 0.39; see Table 2). Results were therefore pooled for further analysis.

**Table 2:** Breakdown of responses according to type of warm-up phase in the attention attribution task.

Cue	Attentive warm up		Inattentive warm up		FET (*P*)
Body	9/9	100%	8/11	73%	0.22
Head	5/10	50%	6/12	50%	>0.99
Eyes	5/10	50%	6/11	55%	>0.99
Mixed	6/10	60%	4/12	25%	0.39

**Figure 2:**
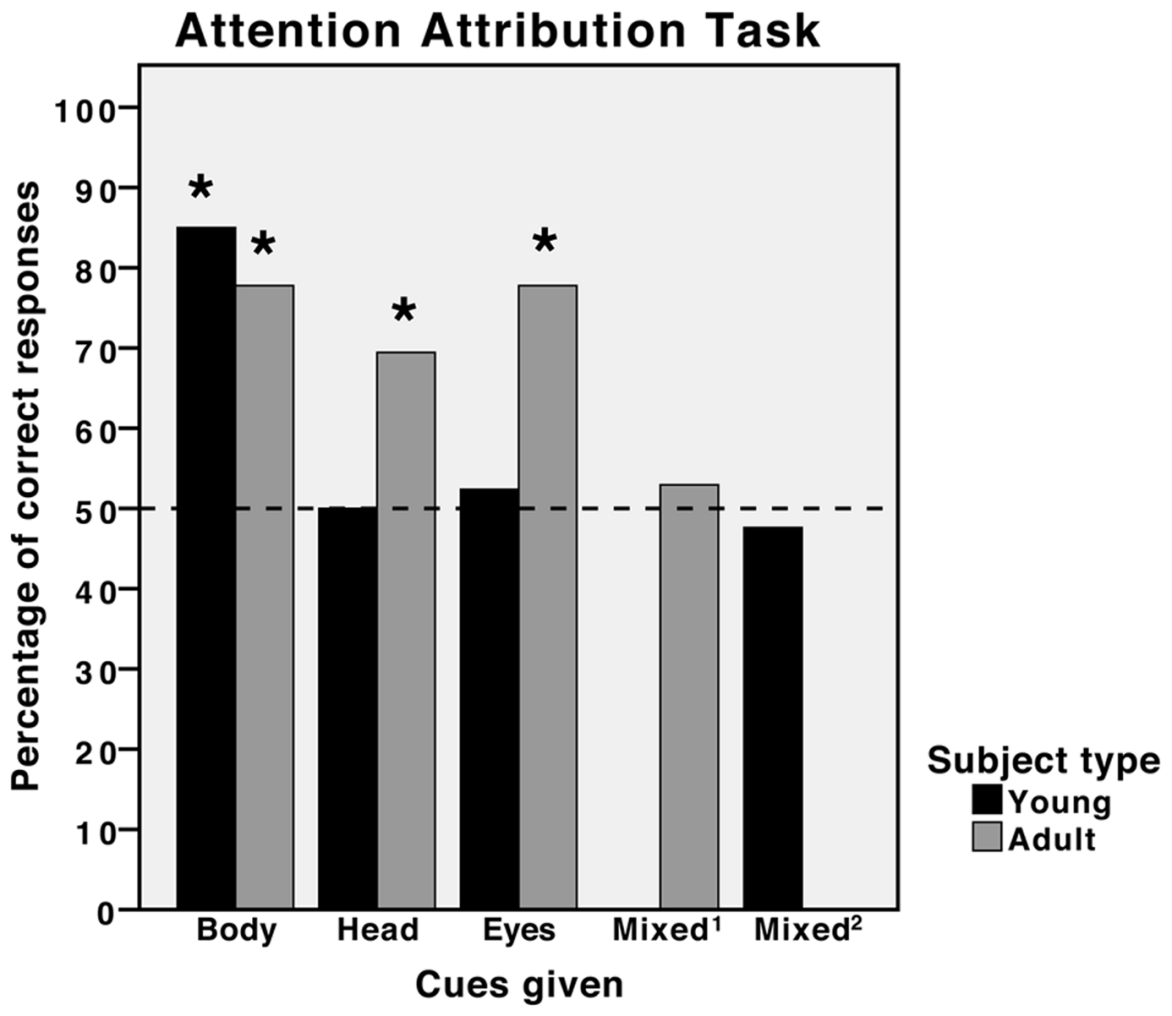
Comparison of the performances of young and adult horses in an attention attribution task. Percentage of correct responses for each cue type for both the youngsters in this study and adult horses reported in the study by Proops and McComb [14]. *  = *p*<0.05 (binomial probabilities, two-tailed predictions). Mixed^1^ refers to the cue given to adult horses in which body and head cues were conflicting. Mixed^2^ refers to the cue given to young horses in which head and eye cues were conflicting.

The scores for each subject can be seen in Table 1. As a group, young horses, like adult horses, chose the attentive person significantly more often than the inattentive person using the body cue (Binomial: *N* = 20, *K* = 17, *P* = 0.003), but unlike adult horses they did not use the head cue (Binomial: *N* = 22, *K* = 11, *P*>0.99) or the eye cue (Binomial: *N* = 21, *K* = 11, *P*>0.99). Neither did the young horses use the mixed cue (Binomial: *N* = 22, *K* = 10, *P*>0.83). The young horses performed at a comparable level to that of adult horses given the body cue (Fisher's Exact: *N* = 56, *P* = 0.39). Although the adult horses were able to use the head cue and the young horses were not, their performances were not found to be significantly different (*χ2*: *N* = 58, *χ2*_1_  = 2.19, *P* = 0.14). The young horses were significantly worse than adult horses at using the eye cue (*χ2*: *N* = 57, *χ2*_1_  = 4.0, *P* = 0.047). See Figure 2 for a comparison of the adult and young horses' cue use in the attention attribution task.

There was no significant difference in the total scores of the 3 age groups (Kruskal-Wallis: *χ2*_2_  = 0.43, *P* = 0.81) nor were there any significant differences in individual cue use based on age (Fisher's Exact Tests: body cue: *N* = 20, *P* = 0.31; head cue: *N* = 22, *P* = 0.75; eye cue *N* = 21, *P* = 0.64, mixed cue: *N* = 22, *P* = 0.55; see Table 3). In addition, there was no significant difference in the overall performance of subjects according to their size (Kruskal-Wallis: *χ2*_2_  = 1.96, *P* = 0.38) nor were there any significant differences in the number of subjects correctly choosing each individual cue based on size (Fisher's Exact Tests: body cue: *N* = 20, *P*>0.99; head cue: *N* = 22, *P*>0.99; eye cue *N* = 21, *P* = 0.48, mixed cue: *N* = 22, *P*>0.99; see Table 4). There was no significant difference between the performance of males and females (Mann-Whitney: *N*_1_ = 12, *N*_2_ = 10, *U* = 50.0, *P* = 0.54). Overall subjects chose the person on their right side more often than the person on their left side (One-sample t test: *N* = 22, *t* = 2.51, *P* = 0.02). At an individual level, four subjects chose the right side for all trials and one subject chose the left side for all trials.

**Table 3:** Breakdown of responses according to age in the attention attribution task.

Cue	0–1yrs		1–2yrs		2–3yrs		FET
	correct	incorrect	correct	incorrect	correct	incorrect	(*P*)
Body	5	2 + 1NR	4	1	8	1NR	0.31
Head	3	5	3	2	5	4	0.75
Eyes	5	3	3	2	3	5 + 1NR	0.64
Mixed	5	3	2	3	3	6	0.554

Number of subjects correctly choosing the attentive person are given by age group. NR  =  no response given. The P values for these accuracy rates, as calculated using 2×3 Fisher's Exact Tests (FET), are reported.

**Table 4:** Breakdown of responses according to size in the attention attribution task.

Cue	<115cm		115–130cm		>130cm		FET
	correct	incorrect	correct	incorrect	correct	incorrect	(*P*)
Body	5	1 + 2NR	7	1	5	1	>0.99
Head	4	4	4	4	3	3	>0.99
Eyes	3	5	5	2 + 1NR	3	3	0.48
Mixed	3	5	4	4	3	3	>0.99

Number of subjects correctly choosing the attentive person are given by size group. NR  =  no response given. The P values for these accuracy rates, as calculated using 2×3 Fisher's Exact Tests (FET), are reported.

### Object choice tasks

The scores for each subject can be seen in Table 1. In the first object choice task, as a group, young subjects, like adult horses, were able to use the distal sustained pointing cue (Binomial: *N* = 25, *K* = 18, *P* = 0.043) but did not use the tapping cue (Binomial: *N* = 25, *K* = 12, *P*>0.99), the body orientation cue (Binomial: *N* = 25, *K* = 11, *P* = 0.69) or the gaze alternation cue (Binomial: *N* = 25, *K* = 13, *P*>0.99). The young horses were also unable to use the elbow point cue (Binomial: *N* = 25, *K* = 16, *P* = 0.23). Of the 18 subjects that correctly used the pointing cue, only 5 investigated the outstretched arm before moving to the bucket compared to 14/23 reported for adults, a significant difference in behaviour (*χ2*: *N* = 41, *χ2*_1_  = 4.45, *P* = 0.035).

**Figure 3:**
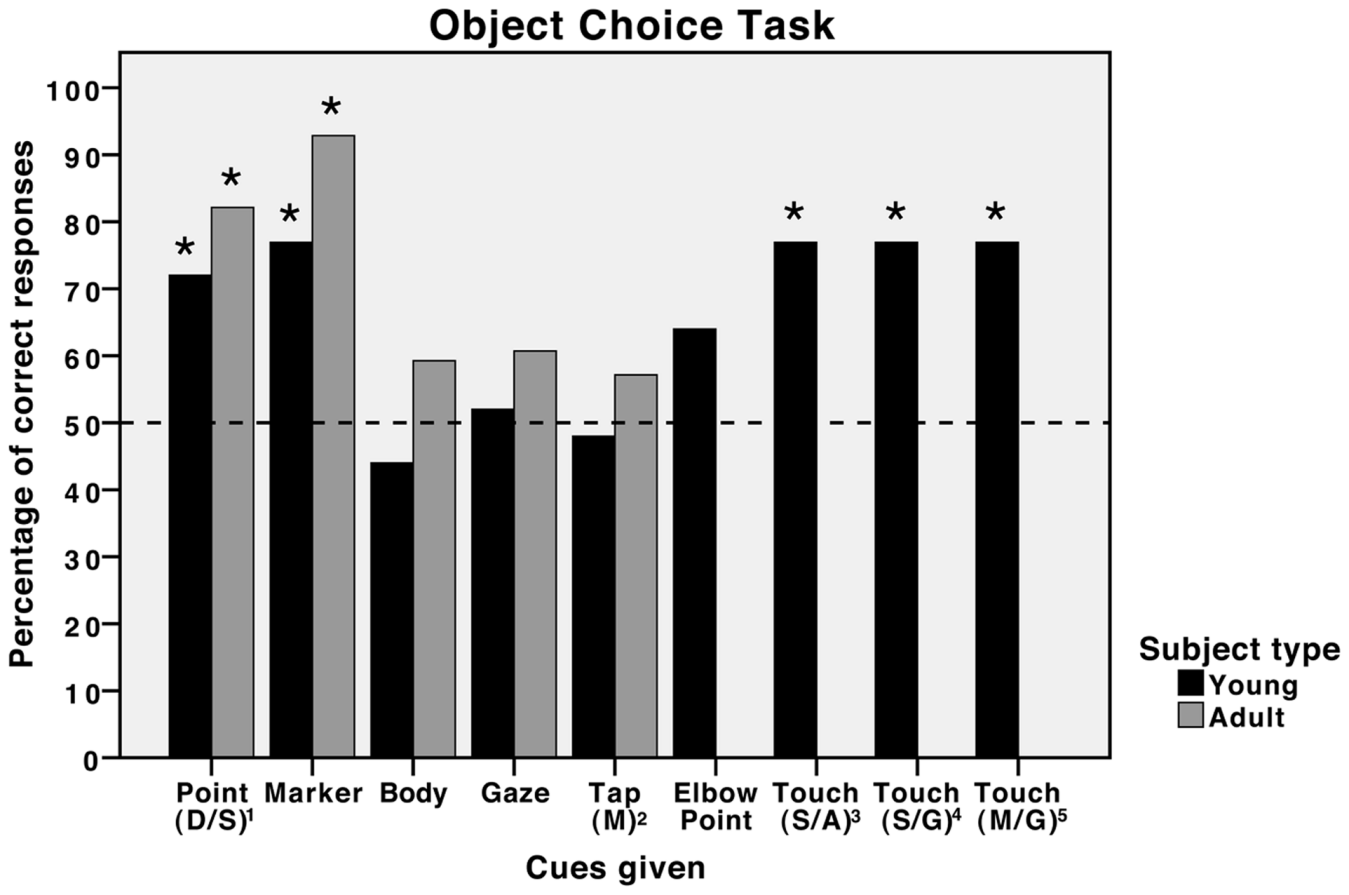
Comparison of the performances of young and adult horses in object choice tasks. Percentage of correct responses for each cue type for both the youngsters in Experiments 1 and 2 from this study and the adult horses reported in the study by Proops et al. [31]. *  = *p*<0.05 (binomial probabilities, two-tailed predictions). ^1^ refers to the distal sustained point cue; ^2^ refers to the momentary tapping cue; ^3^ refers to the sustained touch cue, facing ahead; ^4^ refers to the sustained touch cue, facing the ground; ^5^ refers to the momentary touch cue, facing the ground.

In the second object choice experiment, the young horses as a group were able to use all the cues given; the marker placement cue (Binomial: *N* = 15, *K* = 12, *P* = 0.035), the sustained touch cue, gazing at the ground (Binomial: *N* = 15, *K* = 12, P = 0.035), the momentary touch cue, gazing at the ground (Binomial: *N* = 15, *K* = 12, P = 0.035) and the sustained touch cue, gazing ahead (Binomial: *N* = 15, *K* = 12, P = 0.035). All of the adult horses and 10/12 of the young horses that correctly used the marker placement cue investigated the marker before the bucket. There were no significant differences in the performance of the adult and young horses when given the same cues (*χ2* and Fisher's Exact Test: distal sustained point: *N* = 53, *χ2*_1_  = 0.78, *P* = 0.38; tapping: *N* = 53, *χ2*_1_  = 0.44, *P* = 0.51; body orientation: *N* = 52, *χ2*_1_  = 1.21, *P* = 0.27; gaze alternation: *N* = 53, *χ2*_1_  = 0.41, *P* = 0.52; marker placement: *N* = 43, *P* = 0.32). See Figure 3 for a comparison of the adult and young horses' cue use in the object choice task.

In the first object choice experiment, there was no significant difference in the total scores of the 3 age groups (Kruskal-Wallis: *χ2*_2_  = 1.58, *P* = 0.45) nor were there any significant differences in individual cue use based on age (Fisher's Exact Tests: point cue: *N* = 25, *P* = 0.07; elbow cue: *N* = 25, *P* = 0.68; tap cue *N* = 25, *P* = 0.88, body cue: *N* = 25, *P*>0.99; gaze cue: *N* = 25, *P* = 0.88; see Table 5). There was no significant difference in the overall performance of subjects according to size (Kruskal-Wallis: *χ2*_2_  = 0.83, *P* = 0.66) nor were there any significant differences in individual cue use based on size (Fisher's Exact Tests: point cue: *N* = 25, *P* = 0.74; elbow cue: *N* = 25, *P* = 0.16; tap cue *N* = 25, *P* = 0.88, body cue: *N* = 25, *P*>0.35; gaze cue: *N* = 25, *P* = 0.88; see Table 6). There was no significant difference between the performance of males and females in either experiment (Mann-Whitney: first object choice experiment: *N*_1_ = 14, *N*_2_ = 11, *U* = 69.0, *P* = 0.64, second object choice experiment: *N*_1_ = 7, *N*_2_ = 8, *U* = 24.5, *P* = 0.63). Subjects showed no overall side bias in either object choice experiment (One-sample t test: *N* = 25, *t* = 1.52, *P* = 0.14; *N* = 15, *t* = 0.001, *P*>0.99). At an individual level, 6 subjects chose the right hand bucket in all trials and 1 subject chose the left hand bucket in all trials in the first object choice experiment. All subjects in the second object choice experiment chose each side at least once.

**Table 5:** Breakdown of responses according to age in object choice task 1.

Cue	0–1yrs		1–2yrs		2–3yrs		FET
	correct	incorrect	correct	incorrect	correct	incorrect	(*P*)
Point	7	1	3	5	8	1	0.07
Elbow	4	4	6	2	6	3	0.68
Tap	4	4	3	5	5	4	0.88
Body	3	5	4	4	4	5	>0.99
Gaze	4	4	5	3	4	5	0.88

Number of subjects correctly choosing the rewarded bucket are given by age group. NR  =  no response given. The P values for these accuracy rates, as calculated using 2×3 Fisher's Exact Tests (FET), are reported.

**Table 6:** Breakdown of responses according to size in object choice task 1.

Cue	<115cm		115–130cm		>130cm		FET
	correct	incorrect	correct	incorrect	correct	incorrect	(*P*)
Point	8	2	6	3	4	2	0.74
Elbow	4	6	7	2	5	1	0.16
Tap	4	6	5	4	3	3	0.88
Body	5	5	5	4	1	5	0.35
Gaze	6	4	4	5	3	3	0.88

Number of subjects correctly choosing the rewarded bucket are given by size group. NR  =  no response given. The P values for these accuracy rates, as calculated using 2×3 Fisher's Exact Tests (FET), are reported.

## Discussion

### Attention attribution task

In the attention attribution task young subjects, at the group level, could use the gross cue of body orientation to determine whether someone was paying attention to them but, unlike adults horses, could not use the more subtle cues of head direction and eye gaze [43]. It is unlikely that the reduced performance of the juveniles is due to a lack of motivation as all subjects were willing to approach the human experimenters and an overall reduction in performance rather than a reduction in performance of specific cues would be evident if there were attentional or motivational causes. In such a precocial species it is also unlikely that the youngsters' lack of cue use reflects any maturational effects. The results therefore suggest that horses' ability to read human attentional cues, while present at a relatively early age, become refined over time and appear to require significant experience to fully develop.

Our results parallel the findings from other attention attribution and gaze following studies. The ontogeny of gaze following develops in distinct stages in human infants as well as primates and others species. In the case of both humans and macaques, the ability to follow human gaze using head orientation appears before the use of subtle eye cues [7], [22]. This gradual development in the complexity of gaze following skills has been attributed to the learning of more complex social communicative skills during the individual's lifetime rather than reflecting any maturational effect. Similarly, young ravens follow the gaze of humans soon after fledging but can only perform the more complex task of following gaze around a barrier several months later [62]. Moreover, ravens habituated to the simple gaze following task but not to gazes around a barrier, leading the authors to suggest that these may be two functionally different and cognitively complex modes of gaze following. Thus it would appear that basic attention attribution skills, utilising gross bodily cues, are present at an early age in horses and some other species, but the ability to use more subtle cues and to employ them in more complex contexts requires experience to develop.

In contrast to the findings presented here, young horses are able to use subtle head and eye cues when determining whether to obey a learnt command [20]. There are several possible explanations for the difference in our findings. It may be that the ability to use subtle attentional cues in a dominant context is present at an earlier age and is a less cognitively demanding task than using attention attribution skills in a cooperative context. Eye gaze is likely to be a highly salient and functionally important cue to use in the context of aggressive/dominance and predator-prey interactions. In our attention attribution task, the context is more cooperative and subjects may not have been as vigilant as subjects presented with a stranger giving them a command. A number of species have shown greater sensitivity to attentional cues in a predator/competitive context than a cooperative context [29], [30] and while the ability to attend to attentional cues in such negative contexts appears to be widespread, the ability to attend to and share attention in cooperative social contexts appears to be less common [30]. The second possibility is that young horses learnt to use subtle attentional cues during the training phase of the Sankey study whereas our study investigates the untrained abilities of young horses. It would be of interest to assess the extent to which horses explicitly trained to obey a command could transfer their use of subtle attention cues to a more cooperative context and similarly to investigate the untrained abilities of young horses in a dominant rather than cooperative context. This would help to determine whether young horses' sensitivity to subtle attentional cues in a dominant/negative context reflects a different, innate mechanism to the attention attribution mechanism that requires experience to be used in a cooperative context, or whether horses simply have to learn to use the same mechanism and then apply it across a range of different contexts.

Although we found a difference between adult and young horses, in this study we did not find a difference in the performance of the young subjects according to age group. However, the relatively small sample size and the effect of different rearing histories may have masked any potential age effects. Thus a more extensive study with subjects from a standardised rearing environment would be a valuable direction for future research. It is also currently unknown which cues horses use to determine the direction of conspecifics' attention and how this ability develops. It is possible that horses possess an innate ability to read subtle conspecific attentional cues, including eye cues, but must learn to transfer this ability to human behaviour, or even more specifically, to human behaviour in a cooperative context. Since horses have a wide field of vision and do not have white sclera, it is equally possible that head and/or eye cues are not reliable predictors of conspecific attention and horses may rely on more overt cues to attention in conspecifics and learn to use more subtle predictive cues when interacting with people. Another possibility is that horses require experience of both conspecifics as well as heterospecifics in order to use subtle attentional cues. Further research is required to determine which of these possibilities is correct.

As adults, both domestic horses and domestic dogs are highly skilled in the attention attribution task [40], [41], [43]. However, this ability seems to require considerable time to develop fully in horses and in dogs performance is context dependent, appearing to reflect the extent to which dogs had experienced the situation before [40]. Thus it may be that not only horses but also dogs require significant exposure to human behaviour to fully acquire this skill. It would therefore be of interest to test young puppies in this task to determine the extent to which learning is a factor in the development of this skill. It must be noted however, that young puppies tend to have much greater exposure to human behaviour than young horses. Many horses are essentially left with minimal training and interaction beyond the provisioning of food for the first year or more of their lives, so even if a relatively small amount of experience with human behaviour is required to learn to use human attentional cues, it may take a number of years for sufficient exposure to occur. In contrast, most very young puppies may well have already had more exposure to human activity than the horses in this study. Thus, in additional to testing young puppies on this task, it would be of interest to attempt to quantify the nature and extent of the interaction between adult and young domestic horses and dogs and their handlers in order to fully appreciate the differences in the relationship they have with humans.

In this study we also found a significant bias in favour of the person on the right side. This may be because the young horses could only use one of the human attentional cues given and so relied on spatial cues instead. It is well know that horses, including foals, readily use spatial cues in learning tasks [63]. It is particularly interesting however, that they consistently chose the right side. Horses have shown lateral biases in information processing across a variety of tasks, preferring to use their right eye when viewing novel objects, their left when viewing a person and their right when identifying familiar people cross-modally [64]–[66]. Lateralisation has not previously been seen in horses during the attention attribution task, presumably because in adult horses the side of (most of) the cues, rather than the spatial configuration itself, was the most salient feature of the task. It is therefore not clear what aspect of the task led to a preference for the right side.

### Object choice tasks

In the object choice task the youngsters performed at a comparable level to adults horses in that, at the group level, they were able to use a distal sustained pointing cue and a marker placement cue but were not able to use the more subtle body orientation and gaze cues [60], [61]. It is also interesting to note that although not significant, 64% of subjects chose the bucket indicated by the elbow point cue, a cue that may provide weak stimulus enhancement. Given a larger sample size it may become evident that young horses can use this cue at a significant level. Neither the juvenile horses tested here nor the adult horses tested by us previously were able to use the momentary tapping cue. Initially this finding was surprising because the cue also provides some stimulus enhancement, although not when the choice is made. The fact that horses have been shown previously to be able to use a proximal momentary pointing cue and in this study have been shown to use a momentary touching cue [61], suggests that it is not the delay between the administering of the cue and the choice being made that leads to the cue not being used. Our results suggest that it is the large arm movements that may have discouraged some of the horses from approaching the bucket given the tapping cue from these studies.

Although the difference in the performance of the youngsters and adults given the pointing cue was not significant (youngsters: 72%; adult: 82%), there was a difference in their behaviour. Only 29% of young subjects that used the pointing cue investigated the hand before the bucket whereas in the adult study 61% investigated the hand. This seems to suggest that the young horses had not yet formed a strong association between the human hand and the provisioning of food and that this association may serve to improve performance in these tasks when pointing cues are used. This is also a factor that has been suggested to contribute to the performance of domestic dogs in this task [67]. All the adult horses and 10/12 of the young horses that correctly used the marker cue, investigated the marker before the bucket, strongly suggesting that the object provided local enhancement for both groups.

There may still be some maturation of this skill beyond three years of age but the use of the sustained pointing and marker placement cues by young horses suggests that their use requires little (or even no) experience of humans to develop. The nature of the cues used suggests that horses tend to rely on stimulus enhancement, a basic and generalised learning mechanism that is possessed by many species and as such it is perhaps not surprising that this skill appears to be present at a relatively early age. The young of all species tested to date (including domestic dogs, hand reared wolves and socialized fox cubs – both those that are selected for tameness and those that are not) are all able to use basic pointing cues (those that provide a degree of stimulus enhancement), supporting the notion that this ability is widespread and develops at an early age [68], [69]. In contrast, the use of distal momentary pointing appears to be a much more complex skill that is acquired by domestic dogs around 2 months of age [70], is not seen in juvenile wolves, only rarely in highly enculturated adult wolves [55], [68], [71] and has not been seen in adult horses [61]. The results from the studies of adult and young horses therefore suggest that horses have little understanding of the communicative intentions underlying cue production and their limited success in this task is due to a simple and very general learning mechanism rather than a specific enhanced ability to read human-given cues.

### General discussion

Our results suggest that the skills required by horses to perform the two human-reading tasks in our studies require different cognitive mechanisms with different patterns of development. Adult horses are highly skilled at reading subtle human body cues to determine the direction of a person's attention and this is a skill that appears to require significant experience to develop. Although the young of many species are sensitive to eye cues in a predator context, the ability to follow the gaze of others in a social context follows distinct developmental stages in humans and other animals including macaques and ravens, and may require a different, more demanding cognitive mechanism [7], [22], [62]. In these species, basic head orientation cues are used soon after birth but the ability to use subtle human eye cues and to use this ability in more complex situations requires experience to develop. In horses the ability to use these more subtle cues, and potentially to employ them in more complex, social contexts, also requires learning during an individual's lifetime. Thus we find no evidence of an innate predisposition to be skilled at reading human attentional cues, rather the developmental trajectory follows that seen in other species studied.

In contrast to the skilled use of human attentional cues by adult horses, adult and young horses are not particularly skilled at attending to human communicative cues to choose a rewarded container in the object choice task. Horses are only able to use cues that provide stimulus enhancement and this skill is present at a relatively early age. Thus their performance in this task is likely to reflect the use of a very general and simple learning mechanism that does not require any extensive exposure to human behaviour beyond the acceptance of them as social partners.

The early presence of a skill does not necessarily mean it is innate, nor the late onset of an ability mean it is learnt. We are also aware that the horses in this study were not young foals and had received some exposure to humans. However, we could not test subjects until they were several months old because they had to able to eat food rewards, concentrate on a task which takes approximately 15–20 minutes and be sufficiently used to human handling that they could be led around the test area. Despite this, these results strongly suggest that horses' ability to read human attentional cues does not reflect an inherent sensitivity to human cues, rather it is a skill that develops through extensive experience over a horses' lifetime. The more limited ability of horses to use human given-cues, although present at an early age, appears to reflect a general learning mechanism that is evident in a number of wild as well as domestic species in which the individuals tested have accepted humans as social partners [4], [68], [69], [71]. The results of this study do not, therefore, support the notion that domestic horses possess an innate predisposition to be particularly skilled at reading human attentional and communicative cues. By comparing the ontogeny of a wide range of attentional and gestural reading skills across species we can begin to understand the different mechanisms required for such tasks and the environmental and genetic factors which give rise to these abilities.
